# Tropical Sunset Blues

**DOI:** 10.3201/eid2409.AC2409

**Published:** 2018-09

**Authors:** Byron Breedlove, Paul M. Arguin

**Affiliations:** Centers for Disease Control and Prevention, Atlanta, Georgia, USA

**Keywords:** art science connection, emerging infectious diseases, art and medicine, about the cover, Paul Klee, Tropische Dämmerung (Tropical Twilight), Tropical Sunset Blues, malaria, mosquito-borne diseases, vector-borne infections, zoonotic infections, zoonoses, public health

**Figure:**
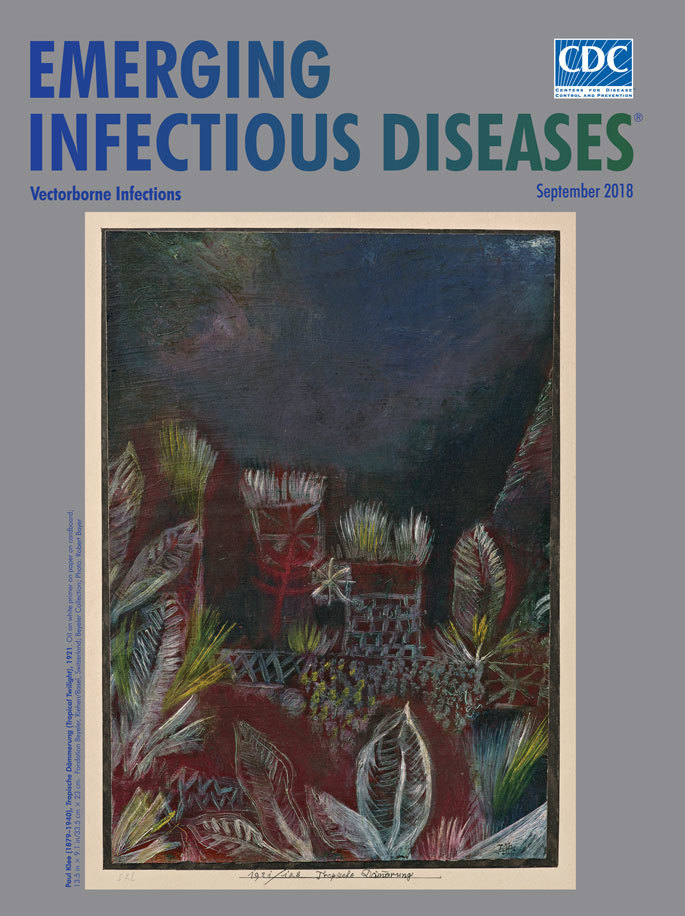
**Paul Klee (1879–1940), Tropische Dämmerung (Tropical Twilight), 1921.** Oil on white primer on paper on cardboard; 13.5 in × 9.1 in/33.5 cm × 23 cm. Fondation Beyeler, Riehen/Basel, Switzerland; Beyeler Collection; Photo: Robert Bayer

“Color is the place where our brain and the universe meet,” according to painter, printmaker, draftsman, and teacher Paul Klee. This month’s cover image, Tropische Dämmerung (Tropical Twilight), is a shimmering example of Klee’s early work during his very productive decade at the German art school Bauhaus. In this painting, Klee uses colors, shapes, and forms that defy expectations for a tranquil twilight in the tropics, in some ways suggestive of the peculiar, imaginary plant kingdom envisioned half a century later in Leo Lionni’s book *Parallel Botany*. Brisk, pale brushstrokes make up the broad leaves and twitching tendrils of vegetation set against a menacing crimson sunset. A crosshatched fence bisects the canvas and is repeated again in the lower left. Hieroglyphic shapes, particularly a starlike symbol, contrast with his organic forms, creating additional tension to the scene. The viewer scans, ruminates, and ponders how Klee’s iconography, arrangement, and color form a seamless and foreboding snapshot of the tropics at that moment when colors fade into darkness.

Klee was born in Münchenbuchsee, Switzerland, in 1879. According to a Tate Museum biography, “Klee came from a generation that would shape the modern world. Albert Einstein . . . was born in March 1879, Georges Braque and Pablo Picasso in 1880 and 1881 respectively.” Klee is remembered as a prodigious artist, caring teacher, talented violinist, and thoughtful writer. His father, Hans Wilhelm Klee, a German music teacher, and his mother, Ida Marie Klee, a Swiss singer, no doubt nurtured his interest and instruction in music, but Klee shifted his attention to visual arts while a teenager and, starting in 1898, he studied drawing and painting in Munich, Germany. Early in his career, Klee struggled to find his path as an artist, noting after a trip to Italy “that a long struggle lies in store for me in this field of color.”

When he returned to Munich in 1911, Klee became involved with Der Blaue Reiter (The Blue Rider), an organization of diverse artists founded by Wassily Kandinsky and Franz Marc. Those associations and travels to Paris in 1912 exposed the young artist to emerging, new theories about color and art forms and lead to his discovery of work by Pablo Picasso and Georges Braque. It was during a brief excursion to Tunisia in 1914, however, that Klee experienced an epiphany about color, attributed to the quality of light there, leading him to boldly proclaim, “Colour has taken possession of me; no longer do I have to chase after it, I know that it has hold of me forever... Colour and I are one. I am a painter.” Klee taught at the Bauhaus from 1921 to 1931, and in 1923, Kandinsky and Klee formed Die Blaue Vier (The Blue Four). 

His influences and his output reveal a gracious fluidity in his vast trove of artwork, thought to comprise 9,000–10,000 works. Identifying Klee by a single category or school of art—whether cubist, abstract, surrealist, expressionist, or perhaps Dadaist—is simply not possible. Klee worked simultaneously on multiple projects in various media, and those could also be quite dissimilar in style and approach. (Klee was also ambidextrous: he used his left hand to paint, his right hand to write.) Alexxa Gotthardt, staff writer and editor for *Arsty,* explains that “Klee’s body of work isn’t easily bucketed into a single category, thanks in large part to the system of throbbing forms, mystical hieroglyphs, and otherworldly creatures that he developed to populate his compositions.”

Whether Klee’s rendering of a tropical twilight came from a place he visited or imagined does not matter. But Klee asserted in his 1920 *Creative Confession* that “Art does not reproduce the visible, rather, it makes visible.” We see the tropical twilight through his eyes as both the natural beauty and the hidden dangers together.

It is at this juncture between day and night when crepuscular fauna are active and the stealthy nocturnal denizens of the tropics begin to stir. Before you can see them, the sting of that first sandfly bite is often the signal that you have lingered too long watching the sunset and it is time to head inside for the evening. Tiny female *Anopheles* mosquitoes also become active at this time, seeking a blood meal for sustenance while perpetuating the devastating cycle of malaria infections. Alphonse Laveran, the scientist who discovered the malaria parasite *Plasmodium falciparum,* died in 1922, the year after Klee finished this arresting painting that evokes the world’s tropical areas. By 2050, according to the report *State of the Tropics,* more than half of the world’s population and 60% of children will live in the tropics. Within the tropics, malaria, the World Health Organization’s 17 neglected tropical diseases, and numerous other zoonotic infections such as leptospirosis and human trypanosomiasis are leading causes of death and disability in humans. 
